# Profiling of Biomarkers for the Exposure of Polycyclic Aromatic Hydrocarbons: Lamin-A/C Isoform 3, Poly[ADP-ribose] Polymerase 1, and Mitochondria Copy Number Are Identified as Universal Biomarkers

**DOI:** 10.1155/2014/605135

**Published:** 2014-07-10

**Authors:** Hwan-Young Kim, Hye-Ran Kim, Min-Gu Kang, Nguyen Thi Dai Trang, Hee-Jo Baek, Jae-Dong Moon, Jong-Hee Shin, Soon-Pal Suh, Dong-Wook Ryang, Hoon Kook, Myung-Geun Shin

**Affiliations:** ^1^Department of Laboratory Medicine, Chonnam National University Medical School and Chonnam National University Hwasun Hospital, 160 Ilsimri, Hwasun-eup, Hwasun-gun, Jeollanam-do 519-809, Republic of Korea; ^2^Brain Korea 21 Plus Project, Chonnam National University Medical School, Gwangju, Republic of Korea; ^3^Laboratory of Metabolism, National Cancer Institute, National Institutes of Health, Bethesda, MD, USA; ^4^Environment Health Center for Childhood Leukemia and Cancer, Chonnam National University Hwasun Hospital, Hwasun, Republic of Korea

## Abstract

This study investigated the profiling of polycyclic aromatic hydrocarbon- (PAH-) induced genotoxicity in cell lines and zebrafish. Each type of cells displayed different proportionality of apoptosis. Mitochondrial DNA (mtDNA) copy number was dramatically elevated after 5-day treatment of fluoranthene and pyrene. The notable deregulated proteins for PAHs exposure were displayed as follows: lamin-A/C isoform 3 and annexin A1 for benzopyrene; lamin-A/C isoform 3 and DNA topoisomerase 2-alpha for pentacene; poly[ADP-ribose] polymerase 1 (PARP-1) for fluoranthene; and talin-1 and DNA topoisomerase 2-alpha for pyrene. Among them, lamin-A/C isoform 3 and PARP-1 were further confirmed using mRNA and protein expression study. Obvious morphological abnormalities including curved backbone and cardiomegaly in zebrafish were observed in the 54 hpf with more than 400 nM of benzopyrene. In conclusion, the change of mitochondrial genome (increased mtDNA copy number) was closely associated with PAH exposure in cell lines and mesenchymal stem cells. Lamin-A/C isoform 3, talin-1, and annexin A1 were identified as universal biomarkers for PAHs exposure. Zebrafish, specifically at embryo stage, showed suitable *in vivo* model for monitoring PAHs exposure to hematopoietic tissue and other organs.

## 1. Introduction

Polycyclic aromatic hydrocarbons (PAHs) are ubiquitous environmental toxicants found in air, water, plants, and soils which are present as volatile, semivolatile, and particulate pollutants [1]. PAHs have been of increasing concern in the human health field due to their wide-spread dispersion in the environment and the adverse health effects associated with PAHs exposure such as carcinogenesis and endocrine disruption. Although the adverse effects of individual PAHs are not exactly alike, the United States Environmental Protection Agency (EPA) has designated 32 PAHs compounds as priority pollutants (http://www.epa.gov/). The toxicity of PAHs is structure dependent. Benzopyrene (BaP) among 32 PAH compounds is notable for being the first chemical carcinogen to be discovered [2, 3].

The most commonly used biomarkers of PAHs exposure are metabolites of PAHs, particularly 1-hydroxypyrene (1-OHP), and PAH-DNA or protein adducts [3]. 1-OHP is the principal product of pyrene metabolism, representing 90% of its metabolites [4]. Following inhalation, the half-life of 1-OHP is on average about 18–20 hours [5–7]. Pyrene is the only known precursor of 1-OHP [8]; it forms a consistent proportion of higher molecular weight PAHs in the environment [9]. Main analytical methods employed to measure 1-OHP are high performance liquid chromatography (LC) combined with fluorescence detection and gas chromatography with mass spectrometry [10, 11].

Biomarkers to assess exposure to PAHs at high levels are well studied, but more work is needed to validate these biomarkers when exposure occurs at low, environmental levels. Most reported biomarkers for PAHs exposure were mainly targeted against nuclear genome and proteome as well as metabolites in either serum or urine. Moreover, biomarkers as mentioned in several studies [3] did not reflect PAHs exposure sensitively in genomic and proteomic level.

Enormous strides have recently been made in our understanding of the biology and pathobiology of mitochondria. Many diseases have been identified as caused by mitochondrial dysfunction, and many pharmaceuticals have been identified as previously unrecognized mitochondrial toxicants. A much smaller but growing reports indicate that mitochondria are also targeted by environmental pollutants [12]. Past evidence had indicated that the mtDNA repair capacity is limited and that the proximity of mtDNA to sites of reactive oxygen species generation suggested that mtDNA may be more susceptible to mutation than nuclear DNA. Our laboratory has recently reported that hnRNP protein and the change of mitochondrial genome are recognized as novel and useful markers for benzene exposure [13]. Moreover, there is currently a paucity of data on the direct effects of PAHs in primary hematopoietic cells and various cell lines.

In zebrafish, hundreds of genes involved in the formation of virtually every organ system have been identified by large-scale mutagenesis screening [14]. Consequently, the phenotypes resulting from loss of gene function through mutation can be compared to malformations resulting from embryonic exposure to contaminants. This “chemical genetic” approach has been used recently to identify specific mechanisms of developmental toxicity [15, 16].

Therefore, this study investigated to identify new biomarkers and pathobiological role for PAHs exposure, especially BaP using targeted mitochondrial genomic and proteomic approach in cell line, peripheral blood/mesenchymal stem cell, and* in vivo* zebrafish model.

## 2. Materials and Methods

### 2.1. Reagents and Cell Lines

Cell lines (K562, THP-1, MOLT-4, and HL-60 cells) were obtained from the American Type Culture Collection, which were cultured in RPMI 1640 medium (Gibco Laboratories, Grand Island, NY, USA) supplemented with 10% fetal bovine serum (Gibco) (see Supplementary Table  1 in Supplementary Material available online at http://dx.doi.org/10.1155/2014/605135). Previous published protocol was used for the isolation and characterization of bone marrow-derived mesenchymal stem cells (h-TERT) [17]. For* in vitro* cell line study, cells were cultured and maintained in RPMI media containing 10% fetal bovine serum and four types of PAHs such as BaP, pentacene, fluoranthene, and pyrene were added in the cell culture media with 100 *μ*M concentration.

### 2.2. Chemicals

BaP (purity > 99%), fluoranthene (99%), pentacene (>99%), and pyrene (>99%) were purchased from Sigma (Sigma-Aldrich, St. Louis, MO, USA). Stock PAHs solutions were made in dimethyl sulfoxide (DMSO) (Sigma-Aldrich) at concentration of 100 *μ*M.

### 2.3. Cytotoxicity Assay

Cytotoxicity assays were carried out using the Enhanced Cell Viability Assay Kit (EZ-CyTox, Daeil Lab Service Co., Seoul, Korea) protocol. The absorbance (A450) of each well was measured using a VERSA Max microplate reader (Molecular Devices, Sunnyvale, CA, USA).

### 2.4. Determination of mtDNA Copy Number

mtDNA copy number was determined according to our published protocol [13]. For* in vitro* model study, the purified PCR product of* cytochrome b (Cytb)* gene was inserted into pGEM-T easy vector and E. coli JM 109 cells (Promega, Madison, WI, USA) were transformed in order to obtain recombinant plasmids. The mtDNA copy number was calculated using the following formula: [*X* *μ*g/*μ*L plasmid DNA/4419 (plasmid length) × 660] × 6.022 × 10^23^ = *Y* molecules/*μ*L, where *X* represents the concentration of plasmid DNA and *Y* represents copy number. For* in vivo* model study, a mixture of 25 *μ*L containing 12.5 *μ*L 2x QuantiTect SYBR green PCR master mix (Qiagen, Valencia, CA, USA), 400 *μ*M* Cytb* primers forward (5′-TTCTGAGGGGCCACAGTAAT-3′) and reverse (5′-GGGGTTATTTGATCCGGTTT-3′), and 50 ng of total DNA were used for the PCR with the CFX96 real-time system (Bio-Rad, Hercules, CA, USA). For PCR, 95°C for 15 minutes was followed by 35 cycles of 20 seconds at 94°C, 30 seconds at 52°C, 30 seconds at 72°C, and a melting reaction with a decrease of 1°C per cycle between 72°C and 92°C.

### 2.5. Direct Sequencing of mtDNA Control Region

This study used a published protocol to amplify and sequence the mtDNA* control region* gene and minisatellites (303 poly C, 16189 poly C and 514 (CA) repeat) [13]. The mtDNA sequences obtained were analyzed using the Revised Cambridge Reference Sequence (http://www.mitomap.org/), Blast2 program (http://www.ncbi.nlm.nih.gov/blast/bl2seq/wblast2.cgi), and the MitoAnalyzer (http://www.cstl.nist.gov/biotech/strbase/mitoanalyzer.html) to identify mtDNA aberrations.

### 2.6. Proteomic Assay of Mitochondria-Rich Cellular Fraction

#### 2.6.1. One-Dimensional SDS-Polyacrylamide Gel Electrophoresis

Briefly, an equal amount of proteins (30 *μ*g) was then separated on NuPAGE 4–12% Bis-Tris Gel (Invitrogen; Carlsbad CA, USA). After separation, the gel was stained with GelCode Blue Stain Reagent (Thermo scientific) and the blue-stained gel lanes were removed by manual cutting. Each blue-stained gel lane was separately cut into 5 slices. Each of these gel slices was then further cut into sizes of ~1 mm^3^ and transferred to a clean 1.5 mL tube.

#### 2.6.2. Enzymatic In-Gel Digestion

The separated proteins were excised from the gel and the gel pieces containing protein were destained with 50% acetonitrile (ACN) containing 50 mM NH_4_HCO_3_ and the gel pieces were vortexed until Coomassie Brilliant Blue was completely removed. These gel pieces were then dehydrated in 100% ACN and vacuum-dried for 20 min with SpeedVac. For the digestion, gel pieces were reduced using 10 mM dithiothreitol in 50 mM NH_4_HCO_3_ for 45 min at 56°C, followed by alkylation of cysteines with 55 mM iodoacetamide in 50 mM NH_4_HCO_3_ for 30 min in the dark. Finally, each of gel pieces was treated with 12.5 ng/*μ*L sequencing grade modified trypsin (Promega) in 50 mM NH_4_HCO_3_ buffer (pH 7.8) at 37°C overnight. Following digestion, tryptic peptides were extracted with 5% formic acid in 50% ACN solution at room temperature for 20 min. The supernatants were collected and dried with SpeedVac. Resuspended samples in 0.1% formic acid were purified and concentrated using C18 ZipTips (Millipore, Billerica, MA, USA) before mass spectrometry (MS) analysis.

#### 2.6.3. Nano-LC-Electrospray Ionization-MS/MS Analysis

The tryptic peptides were loaded onto a fused silica microcapillary column (12 cm × 75 *μ*m) packed with C18 reversed phase resin (5 *μ*m, 200 Å). LC separation was conducted under a linear gradient as follows: a 3–40% solvent B (ACN containing 0.1% formic acid) gradient (solvent A; DW containing 0.1% formic acid), with a flow rate of 250 nL/min, for 60 minutes. The column was directly connected to linear trap quadropole linear ion-trap mass spectrometer (Finnigan, San Jose, CA, USA) equipped with a nanoelectrospray ion source. The electrospray voltage was set at 1.95 kV, and the threshold for switching from MS to MS/MS was 500. The normalized collision energy for MS/MS was 35% of main radio frequency amplitude and the duration of activation was 30 ms. All spectra were acquired in data-dependent scan mode. Each full MS scan was followed by five MS/MS scans corresponding to the range from the most intense to the fifth intense peaks of full MS scan. Repeat count of peak for dynamic exclusion was 1, and its repeat duration was 30 seconds. The dynamic exclusion duration was set for 180 seconds and the width of exclusion mass was ±1.5 Da.

#### 2.6.4. Database Searching and Validation

The acquired LC-electrospray ionization-MS/MS fragment spectra were searched in the BioWorksBrowser (version Rev. 3.3.1 SP1, Thermo Fisher Scientific Inc.) with the SEQUEST search engines against National Center for Biotechnology Information (http://www.ncbi.nlm.nih.gov/) nonredundant human database.

### 2.7. Quantitative mRNA Expression Study

Total RNA was extracted using the QIAamp RNA Blood Mini kit (Qiagen). Reverse transcription produced cDNAs using Superscript III (Applied Biosystems). The expression of poly[ADP-ribose] polymerase 1 (PARP-1) and lamin A/C (LMNA) mRNA was quantified using QuantiTect SYBR green PCR master mix (Qiagen), PARP-1 forward: 5′-GAGGAAGTAAAGGAAGCCAA-3′, PARP-1 reverse: 5′-CACAACTTCAACAGGCTCT-3′, LMNA forward: 5′-AAGCTTCGAGACCTGGAG-3′, LMNA reverse: 5′-TCCAAGAGCTTGCGGTA-3′, and *β*-actin mRNA as a normalization control. The ΔΔCt method was used to calculate relative changes in gene expression determined by real-time quantitative PCR using CFX96 (Bio-Rad). Normalization was achieved using Ct values of PARP-1 and LMNA mRNA from PAHs-treated cells and *β*-actin mRNA. The ΔCt_calibrator_ value (mean PARP-1 and LMNA—mean *β* actin) was obtained from the mean Ct value of PARP-1 and LMNA mRNA and *β*-actin mRNA from the control cells (*n* = 10). The ΔΔCt value was calculated as ΔCt minus ΔCt_calibrator_. The final relative quantification of PARP-1 and LMNA mRNA was expressed as ΔΔCt.

### 2.8. Western Blot

Extracted protein samples (20 *μ*g per well) were separated on a 12% SDS-Bis-Tris polyacrylamide gel. After transfer, the nitrocellulose membrane was incubated over night with 10 mL of primary antibodies against PARP-1, LMNA (Santa Cruz Biotechnology, Delaware Avenue, CA, USA), and *β*-actin (Santa Cruz Biotechnology) at 4°C. The membrane was then incubated with the appropriate goat anti-mouse IgG antibody (1 : 1000) (Jackson ImmunoResearch Laboratories, West Grove, PA, USA) to detect biotinylated protein markers in 10 mL of blocking buffer with gentle agitation for 1 hour at room temperature. The proteins were visualized using a chemiluminescence detection system (Amersham ECL system, London, UK).

### 2.9. *In Vivo* Study

For* in vivo* model study, zebrafish embryos 30 h after fertilization (hpf) were exposed to BaP at concentrations of 200, 400, 600, 800, and 1000 nM. Seventy embryos were cultured in 40 mL of BaP solution in each petri dish, and there were three replicates for each of the five treatments. Embryos were collected at 54 hpf, 78 hpf, and 102 hpf. Embryos were maintained under the same temperature and pH conditions for the duration of experiments.

## 3. Results

### 3.1. The Change of Cell Morphology

**Figure 1:**
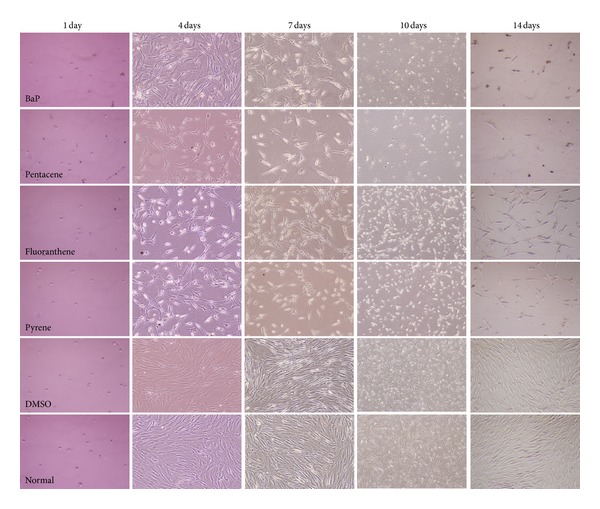
Morphological change of human mesenchymal stem (h-TERT) cells after PAHs exposure. PAH-untreated cells (DMSO and normal) showed compact cellularity with spindle shape. h-TERT cells were tightly attached to each other and to the substrate. Generally, direct exposure of PAHs depressed the proliferative capacity of h-TERT cells with a thread-like or round shape and loose cell-to-cell attachment. Each PAHs compound showed different cytotoxic effect. DMSO and normal indicated only DMSO-treatment and culture solution itself (no treatment of PAHs and DMSO), respectively.

PAH-untreated h-TERT cells showed compact cellularity with spindle shape. Cells were tightly attached to each other and to the substrate. Generally, direct exposure of PAHs such as BaP, pentacene, fluoranthene, and pyrene depressed the proliferative capacity of h-TERT cells and the cell morphology was altered in each PAH-exposure group. Cells became detached from the subsurface, and cell-to-cell attachments were lost (Figure 1).

### 3.2. The Change of Total Cell Counts

**Figure 2:**
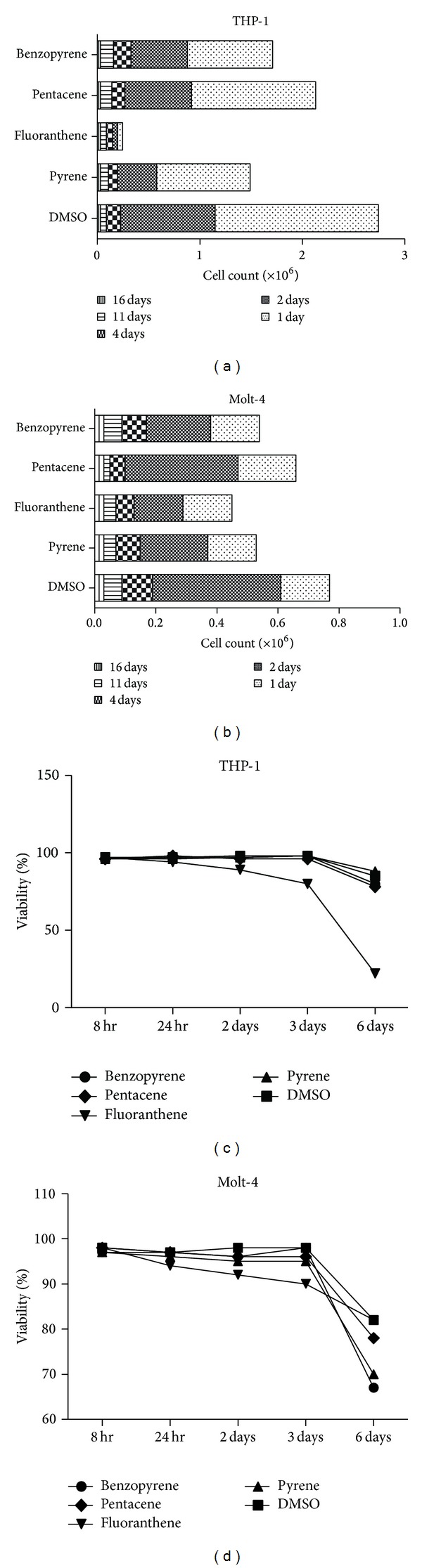
The change of cell count and viability after PAHs exposure in THP-1 and Molt-4 cell line. Depending on the type of PAHs, each cell count showed different aspects. In comparison to DMSO treated (0.1%) group, fluoranthene displayed profound significant reduction in cell count, especially in THP-1 and Molt-4 cell line ((a) and (b)). Viability was significantly decreased after fluoranthene exposure for two days. On the third day of PAHs exposure, viability was reduced remarkably in both cell lines ((c) and (d)).

Depending on the type of PAHs, each cell count showed different aspects. The total number of cells in the THP-1 and Molt-4 cell lines decreased 11 days after PAHs exposure. The change in the total number of cells in the THP-1 and Molt-4 cell lines decreased in a time-dependent manner. In comparison to control group, fluoranthene displayed profound significant reduction in cell count (Figures 2(a) and 2(b)). The change in the total cell count for the THP-1 and Molt-4 cell lines had a similar pattern after PAHs exposure. Cytotoxicity study carried out the experiment with 100 *μ*M of PAHs after selecting the minimum concentration that is poisonous to cells.

### 3.3. Viability and Apoptosis

Viability significantly decreased after two days of exposure to fluoranthene. On the third day of PAHs exposure, viability reduced remarkably in all the cells (Figures 2(c) and 2(d)). Each type of cell lines displayed different proportionality of apoptosis. Several hundreds of PAHs exposure biomarkers were identified in comparison to control group (Supplemental Figure 1).

### 3.4. Increased mtDNA Copy Number

**Figure 3:**
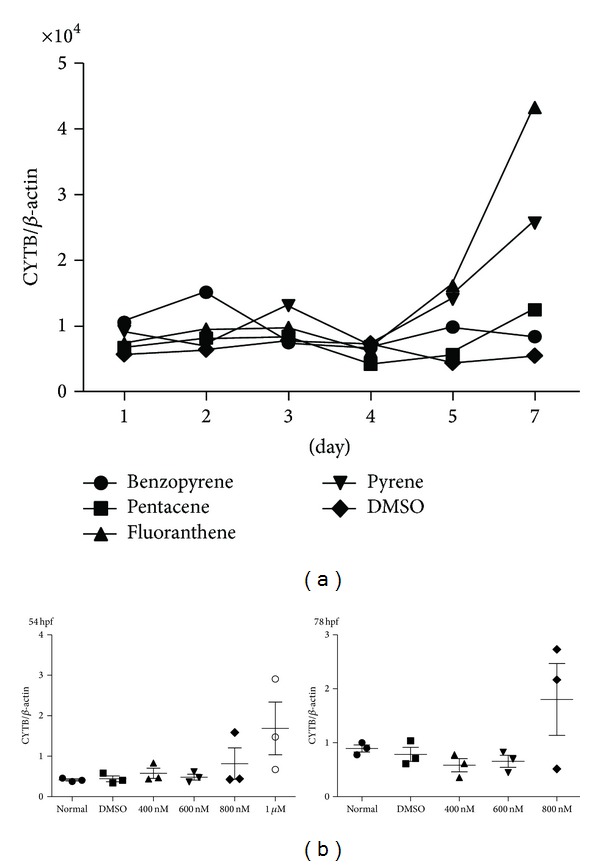
The change of mtDNA copy number after PAHs exposure. mtDNA copy number was increased after exposure of PAHs with different pattern in THP-1 cell line (a) and* in vivo* zebrafish model (b). mtDNA copy number was dramatically elevated after 5-day treatment of fluoranthene and pyrene in both THP-1 cell line and* in vivo* zebrafish model. hpf, hours per fertilization in zebrafish; normal, no treatment group; and DMSO, only DMSO (0.1%) treated group.

Mitochondrial contents were increased with different pattern: mtDNA copy number was dramatically elevated after 5-day treatment of fluoranthene and pyrene in both cell line and* in vivo* zebrafish model. mtDNA copy numbers were generally increased after PAHs exposure in a dose and time-dependent manner in the cell lines. These findings suggested that loss of compensatory ability in response to high levels of oxidative stress was induced by high concentrations of PAHs (Figure 3).

### 3.5. Sequence Alteration of mtDNA Control Region

Changes of the mtDNA sequence were comprehensively studied by direct sequencing of the mtDNA control region and gene scanning for the determination of mtDNA length and heteroplasmic mutations. No alteration of mtDNA sequences was observed after direct exposure of PAHs during 7 days (Supplemental Figure 2). No alteration of mtDNA minisatellites such as 1618 poly C, 303 poly C and 514 (CA) repeat was found after PAHs exposure.

### 3.6. Mitochondrial Protein Markers

**Figure 4:**
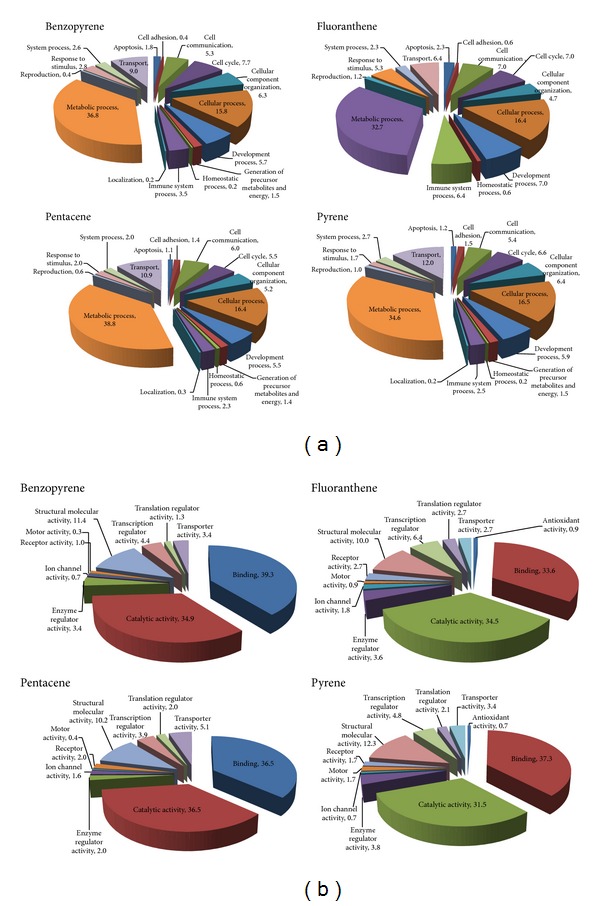
Functional grouping of potential candidate biomarkers for PAHs exposure. Identified potential biomarkers were categorized as their biological process (a) and molecular functions (b). These candidate biomarkers for PAHs exposure were isolated using proteomic analysis of mitochondria-rich cellular fraction in THP-1 cell line.

Several hundreds of cellular proteins in mitochondrial-rich cytoplasmic fraction were profoundly deregulated in comparison to control group (Figure 4). The notable deregulated proteins for PAHs exposure were displayed as follows: LMNA and annexin A1 for BaP; LMNA and DNA topoisomerase 2-alpha for pentacene; PARP-1 for fluoranthene; and talin-1 and DNA topoisomerase 2-alpha for pyrene (Tables 1 and 2).

### 3.7. Confirmation of Mitochondrial Protein Markers 

#### 3.7.1. Increased mRNA Expression of PARP-1 and LMNA Gene

**Figure 5:**
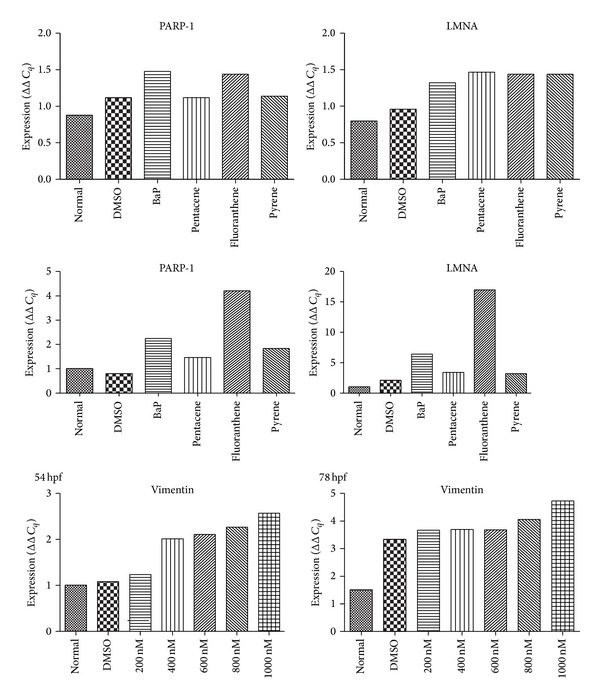
mRNA expression study of candidate biomarker genes. mRNA expression of PARP-1 and LMNA gene was generally increased in THP-1 and h-TERT cell lines after exposure of PAHs with different pattern. Normal, no treatment group; DMSO, only DMSO (0.1%) treated group.

mRNA expression of PARP-1 and LMNA gene was generally increased in THP-1 and h-TERT cell lines after exposure of PAHs with different pattern. This finding was confirmed using embryogenesis in zebrafish model (Figure 5).

#### 3.7.2. Increased Protein Expression of PARP-1 and LMNA Gene

**Figure 6:**
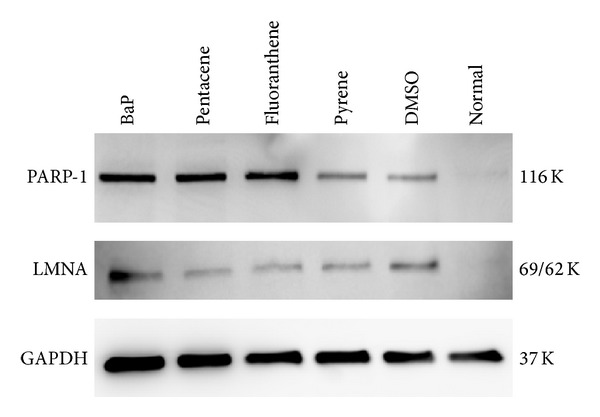
Confirmation of PARP-1 and LMNA biomarkers using Western blot. The expression of PARP-1 was remarkably increased after exposure of BaP, pentacene, and fluoranthene (100 *μ*M concentration). LMNA protein was highly expressed after BaP exposure. Normal, no treatment group; DMSO, only DMSO (0.1%) treated group; K, kilodalton.

The expression of PARP-1 protein was increased after exposure of BaP, pentacene, and fluoranthene. The LMNA proteins were increased after exposure of BaP (Figure 6).

### 3.8. Morphological Abnormalities of Zebrafish

**Figure 7:**
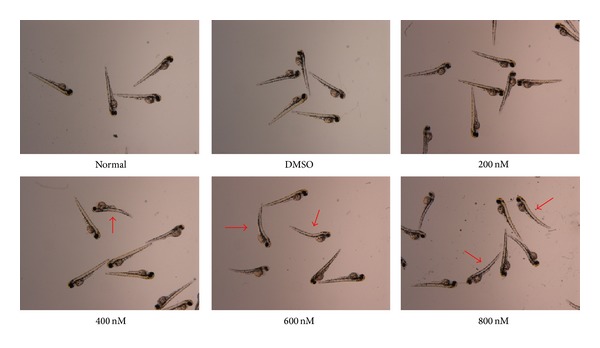
Morphological abnormalities in the general shape of zebrafish after BaP exposure. Obvious morphological abnormalities including curved backbone (arrow) were developed after exposure of more than 400 nM concentration of BaP during the embryogenesis (54 hours per fertilization).

At 54 hpf, embryos treated with 400 nM BaP exhibited mild pericardial edema and showed dorsal curvature of the body axis (Figure 7). Dorsal curvature was more severe by higher concentration of BaP as edema accumulated. Notably, eye and jaw growths were similarly reduced by BaP treatment.

## 4. Discussion

PAHs are known genotoxic agents and induce DNA damaging effects, such as DNA adducts, DNA strand breaks, chromosomal aberrations, sister chromatid exchanges, and micronucleus formation [18]. The main sources of human exposure to PAHs are occupation, passive and active smoking, and food, water, and air pollution [19]. The total intake of carcinogenic PAHs in the general population has been estimated to be 3 *μ*g/day [20]. Levels of occupational exposure of BaP, which is one of the main PAHs compounds, vary widely in different industrial activities and job titles, ranging from 0.1 to 48 000 ng/m^3^ [21–23]. In smokers, BaP levels range from 0.5 to 7.8 *μ*g/100 cigarettes when exposure is from mainstream smoke and from 2.5 to 19.9 *μ*g/100 cigarettes when it comes from side-stream smoke. Levels from passive smoking are lower, ranging from 0.0028 to 0.76 *μ*g/m^3^ of BaP [24]. Besides occupational exposure, dietary intake seems to be the most important source of PAHs in nonsmokers [24, 25]. There is a high variation in atmospheric PAHs levels across geographical areas with BaP concentrations ranging from 0.01 to 100 ng/m^3^ BaP [26]. Airborne PAHs are usually analyzed by gas chromatography/mass spectrometry [27, 28] or high performance LC [29–31], mostly from particles collected in a filter after extraction with organic solvents.

In order to exert its deleterious effects, BaP must be bioactivated. The formation of BaP* o*-quinones has been described as one of the BaP activation pathways. Cytochrome P4501A (CYP1A) is able to produce BaP-7, 8 diol that is further oxidized to BaP-7, 8-dione by AKR1A1 [32]. BaP binds to and activates the aryl hydrocarbon receptor (AhR), being metabolized by the cytochrome P4501A, the microsomal epoxide hydrolase, and the glutathione-S-transferase *α* [33]. AhR is a ligand-activated transcription factor involved in the regulation of biological responses to planar aromatic hydrocarbons. AhR ligands have been generally classified into two categories, synthetic or naturally occurring. The first ligands to be discovered were synthetic and members of halogenated aromatic hydrocarbons. Naturally occurring compounds that have been identified as ligands of AhR include derivatives of tryptophan [34, 35]. The major contributors to air PAHs in the urban and suburban atmosphere are mobile sources from diesel and gasoline engines. Emissions from these sources contain mainly benzo(g,h,i)perylene, pyrene, fluoranthene, and phenanthrene [36], so that measuring only BaP as an index substance may result in exposure underestimation [3].

In this study, a broad molecular investigation of the mitochondrial genome and proteome after PAHs exposure showed an increased mtDNA copy number, PARP-1, and LMNA protein, which could be used as biomarkers for exposure of PAHs in cell lines. PAHs directly might cause an increase in the generation of intracellular ROS, subsequently resulting in a change of the mtDNA content, and proteome. The oxidative stress induced by PAHs can lead to an increase in mitochondrial mass and mitochondrial membrane potential. The mitochondrial genome is highly susceptible to DNA damage caused by ROS and mutagens and has higher rates of mutation than does the nuclear genome. In addition, DNA damage persists longer in the mitochondrial genome. The absence of histones that provide packaging and protection of nuclear DNA and the error-prone replication and repair of mitochondrial genes all contribute to the vulnerable nature of mitochondrial DNA [37]. Therefore, the present study targeted the mitochondrial genome and proteome to identify biomarkers associated with PAH exposure. The results of the present study showed that, after PAHs exposure, mtDNA copy number was increased. The increase of mtDNA copy number was thought to compensate for declining respiratory function during the oxidative stress after PAH exposure.

Mitochondria-rich cellular proteome was then studied to determine whether biomarkers associated with exposure of PAHs could be identified. The result showed that PARP-1 and LMNA protein might be a novel universal biomarker associated with exposure of PAHs. PARP is a monomeric protease widely present in the nuclei of most eukaryotic cells that is associated with the occurrence and development of a variety of diseases. PARP-1, the best characterized member of the PARP family, which currently comprises 18 members, is an abundant nuclear enzyme implicated in cellular responses to DNA injury provoked by genotoxic stress. PARP is involved in DNA repair and transcriptional regulation and is now recognized as a key regulator of cell survival and cell death as well as a master component of a number of transcription factors involved in tumor development and inflammation. PARP becomes activated in response to oxidative DNA damage and depletes cellular energy pools, thus leading to cellular dysfunction in various tissues. The activation of PARP may also induce various cell death processes and promotes an inflammatory response associated with multiple organ failure [38]. It is known to activate nuclear factor-*κ*B (NF-*κ*B) through a variety of pathways, which can lead to increased expression of NF-*κ*B-dependent genes such as oncogenes, cell adhesion molecules, matrix metalloproteinases, and growth factors [39]. Inhibition of PARP activity is protective in a wide range of inflammatory and ischemia-reperfusion-associated diseases, including cardiovascular diseases, diabetes, rheumatoid arthritis, endotoxic shock, and stroke [38]. LMNA, nuclear intermediate filament proteins, is a basic component of the nuclear lamina. Mutations in LMNA are associated with a broad range of laminopathies, congenital diseases affecting tissue regeneration, and homeostasis. This study showed global profiling of toxic changes of PAHs in cell lines, h-TERT, and zebrafish model. The change of mitochondrial genome (increased mtDNA copy number and mass) was closely associated with PAHs exposure in hematopoietic and mesenchymal stem cells. Among cellular proteins, LMNA, talin-1, and annexin A1 were remarkably elevated after exposure of PAHs; these may play a role as biomarkers for PAHs exposure. In zebrafish embryos, we observed pericardial edema and dorsal curvature of the body axis associated with BaP. Zebrafish, specifically embryo stage, showed suitable* in vivo* model for monitoring BaP exposure to hematopoietic tissue and other organs.

## 5. Conclusions

Direct exposure to PAHs induced alteration of the mitochondrial genome including increased mtDNA copy number. The proteomic analysis of the mitochondria-rich cellular fraction showed that PARP-1 and LMNA were a novel universal biomarker associated with exposure of PAHs. Thus mtDNA copy number, PARP-1, and LMNA protein might be useful biomarkers associated with PAHs toxicity and hematotoxicity.

## Supplementary Material

Supplemental Table 1 shows the cell lines used in this study. It provides origin of species, morphology, histopathology and culture medium of each cell lines (K562, THP-1, MOLT-4, HL-60 cells and h-TERT).
Supplemental Figure 1 disloses cytotoxic effect of PAHs compounds on THP-1 cell line. Each PAHs showed different degree of cytotoxic effect, but they showed it with dose-dependent manner.
Supplemental Figure 2 shows mtDNA sequence polymorphisms in zebrafish. No sequence change of mtDNA control region was found after BaP exposure.

## Figures and Tables

**Table 1:** Summary list of identified potential biomarkers for PAHs exposure.

PAHs	Protein	Fold change
Benzopyrene	Vimentin	19.39
	Annexin A1	13.38
	Lamin-A/C	10.04
	NADPH: adrenodoxin oxidoreductase, mitochondrial isoform 2 precursor	5.3
	Squalene synthase	5.3
	Heterogeneous nuclear ribonucleoproteins A2/B1 isoform B1	4.82
	T-complex protein 1 subunit theta	4.35
	Talin-1	4.35

Pentacene	Lamin-A/C	6.9
	DNA topoisomerase 2-alpha	6.38
	Annexin A1	6.38
	Poly[ADP-ribose] polymerase 1	5.85
	Squalene synthase	5.33
	Talin-1	5.33
	PREDICTED: u5 small nuclear ribonucleoprotein 200 kDa helicase-like, partial	4.81

Fluoranthene	Poly[ADP-ribose] polymerase 1	6.21
	Elongation factor 1-gamma	5.21
	Heat shock 70 kDa protein 1A/1B	5.21
	Heterogeneous nuclear ribonucleoproteins A2/B1 isoform B1	5.21
	Probable ATP-dependent RNA helicase DDX5	5.21
	T-complex protein 1 subunit theta	5.21

Pyrene	Talin-1	16.82
	DNA topoisomerase 2-alpha	8.17
	Filamin-C isoform b	7.16
	E3 SUMO-protein ligase RanBP2	5.65
	CAD protein	5.14
	Poly[ADP-ribose] polymerase 1	5.14

**Table 2:** Results of PARP-1 and LMNA protein by repeat proteomic analysis.

Protein	Fold change	
	First result	Second result
PARP-1 (accession no: 156523968)		
DMSO versus BaP	3.41	3.58
DMSO versus pentacene	5.85	4.31
DMSO versus fluoranthene	6.21	5.34
DMSO versus pyrene	5.14	3.41
LMNA (accession no: 27436948)		
DMSO versus BaP	10.04	4.16
DMSO versus pentacene	No change	0.97
DMSO versus fluoranthene	4.50	3.00
DMSO versus pyrene	4.14	1.80

DMSO, only DMSO-treatment as control; PARP-1, poly[ADP-ribose] polymerase 1; LMNA, lamin A/C; BaP, benzopyrene.
