# Supplementary material for: Profiling of Biomarkers for the Exposure of Polycyclic Aromatic Hydrocarbons: Lamin-A/C Isoform 3, Poly[ADP-ribose] Polymerase 1, and Mitochondria Copy Number Are Identified as Universal Biomarkers
Source: Biomed Res Int. 2014 Jul 10;2014:605135. doi: 10.1155/2014/605135 (PMC4121044; doi:10.1155/2014/605135)
Supplement: Supplementary file 1 — Supplemental Table 1 shows the cell lines used in this study. It provides origin of species, morphology, histopathology and culture medium of each cell lines (K562, THP-1, MOLT-4, HL-60 cells and h-TERT). Supplemental Figure 1 disloses cytotoxic effect of PAHs compounds on THP-1 cell line. Each PAHs showed different degree of cytotoxic effect, but they showed it with dose-dependent manner. Supplemental Figure 2 shows mtDNA sequence polymorphisms in zebrafish. No sequence change of mtDNA control region was found after BaP exposure. [file 605135.f1.doc]

**Supplemental Table 1. Cell lines used in this study**

| **General**  **Name** | **Species** | **Morphology** | **Histopathology** | **Culture medium** |
| --- | --- | --- | --- | --- |
| K-562 | Human  (*Homo sapiens)* | Lymphoblastic | CML  (Chronic myelocytic leukemia) | RPMI1640+10%FBS |
| THP-1 | ＂ | Monocytic | AML  (Acute monocytic leukemia) | ＂ |
| MOLT-4 | ＂ | Lymphoblastic | ALL  (T- lymphoblastic leukemia) | ＂ |
| h-TERT |  | Fibroblast | Bone marrow  Mesenchymal stem cell | DMEM |
