# Supplementary material for: Profiling of Biomarkers for the Exposure of Polycyclic Aromatic Hydrocarbons: Lamin-A/C Isoform 3, Poly[ADP-ribose] Polymerase 1, and Mitochondria Copy Number Are Identified as Universal Biomarkers
Source: Biomed Res Int. 2014 Jul 10;2014:605135. doi: 10.1155/2014/605135 (PMC4121044; doi:10.1155/2014/605135)
Supplement: Supplementary file 2 [file 605135.f2.pdf]

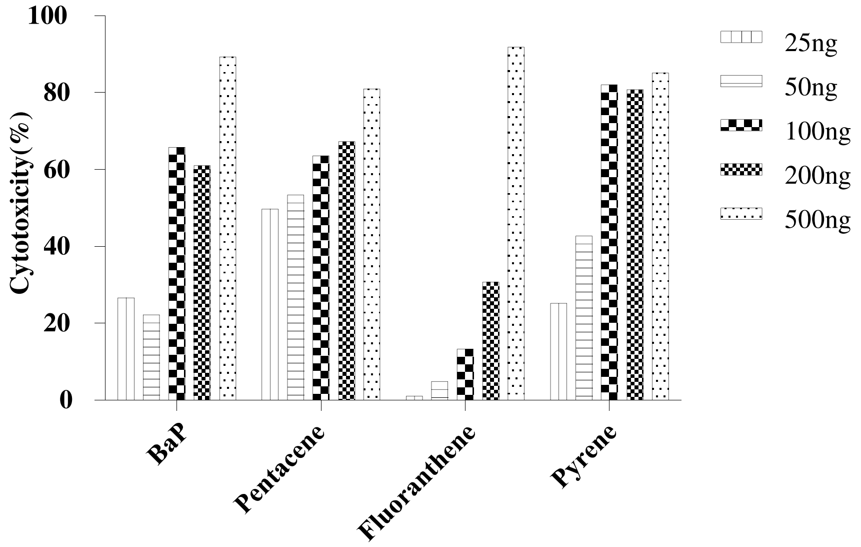


**Supplemental Fig. 1. Cytotoxic effect of PAHs compounds.** Each PAHs disclosed different degree of cytotoxic effect, but they showed it with dose-dependent manner.

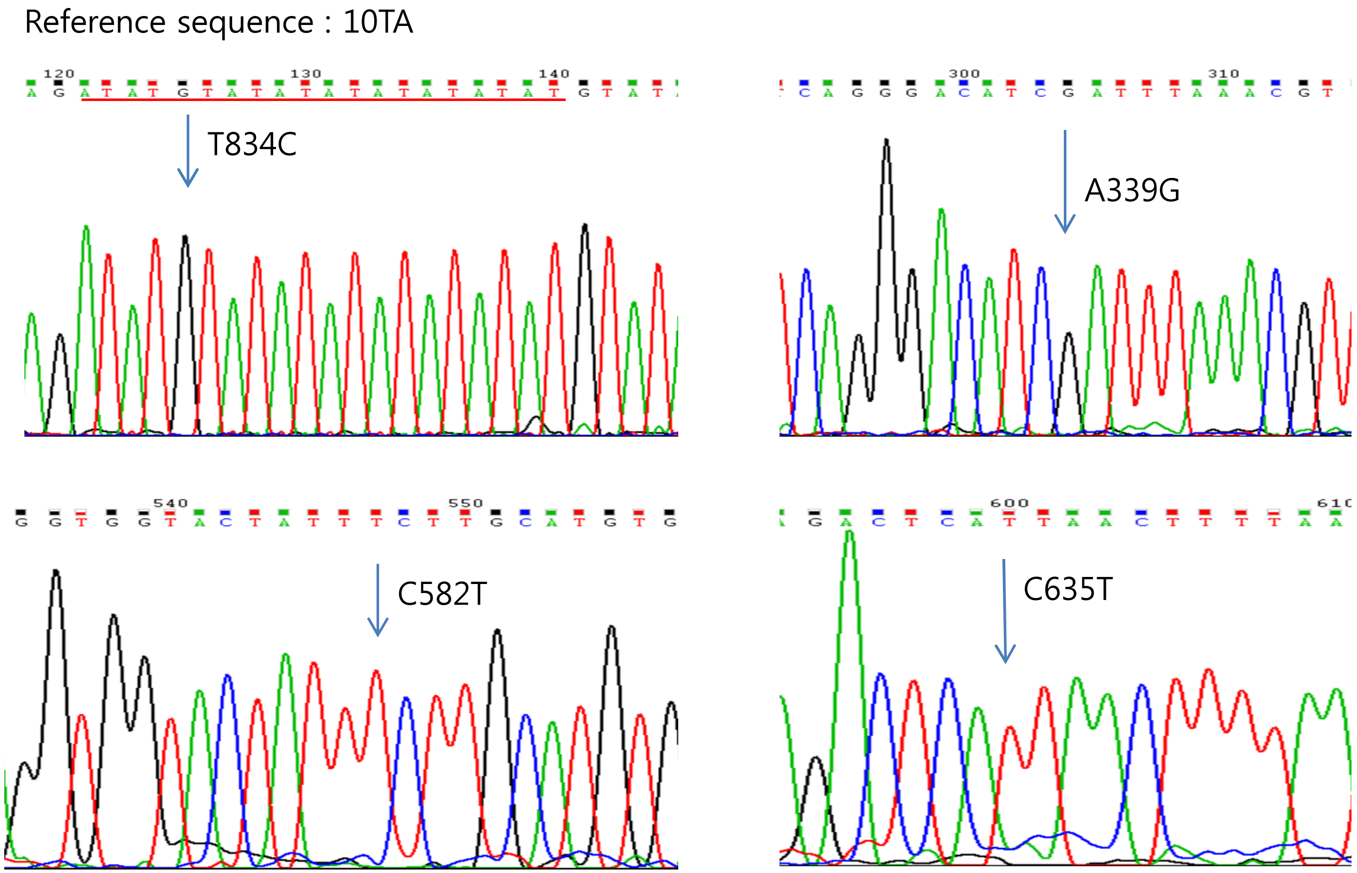


**Supplemental Fig. 2. mtDNA sequence alterations in zebrafish, which assumed polymorphisms.** However, no sequence change of mtDNA control region was found after BaP exposure.
